# A large-scale investigation and identification of methicillin-resistant *Staphylococcus aureus* based on peaks binning of matrix-assisted laser desorption ionization-time of flight MS spectra

**DOI:** 10.1093/bib/bbaa138

**Published:** 2020-07-16

**Authors:** Hsin-Yao Wang, Chia-Ru Chung, Zhuo Wang, Shangfu Li, Bo-Yu Chu, Jorng-Tzong Horng, Jang-Jih Lu, Tzong-Yi Lee

**Affiliations:** 1 Department of Laboratory Medicine, Chang Gung Memorial Hospital at Linkou, Taoyuan City, Taiwan; 2 Department of Computer Science and Information Engineering, National Central University; 3 Warshel Institute for Computational Biology, The Chinese University of Hong Kong, Shenzhen, China; 5 Department of Computer Science & Engineering, Yuan Ze University, Taoyuan City, Taiwan; 7 Department of Computer Science and Information Engineering, National Central University, Taiwan; 8 Warshel Institute for Computational Biology, School of Life and Health Sciences

**Keywords:** Methicillin-resistant *Staphylococcus aureus*, MRSA, mass spectrometry, MALDI-TOF, binning method, machine learning, feature selection

## Abstract

Recent studies have demonstrated that the matrix-assisted laser desorption ionization-time of flight mass spectrometry (MALDI-TOF MS) could be used to detect superbugs, such as methicillin-resistant *Staphylococcus aureus* (MRSA). Due to an increasingly clinical need to classify between MRSA and methicillin-sensitive *Staphylococcus aureus* (MSSA) efficiently and effectively, we were motivated to develop a systematic pipeline based on a large-scale dataset of MS spectra. However, the shifting problem of peaks in MS spectra induced a low effectiveness in the classification between MRSA and MSSA isolates. Unlike previous works emphasizing on specific peaks, this study employs a binning method to cluster MS shifting ions into several representative peaks. A variety of bin sizes were evaluated to coalesce drifted or shifted MS peaks to a well-defined structured data. Then, various machine learning methods were performed to carry out the classification between MRSA and MSSA samples. Totally 4858 MS spectra of unique *S. aureus* isolates, including 2500 MRSA and 2358 MSSA instances, were collected by Chang Gung Memorial Hospitals, at Linkou and Kaohsiung branches, Taiwan. Based on the evaluation of Pearson correlation coefficients and the strategy of forward feature selection, a total of 200 peaks (with the bin size of 10 Da) were identified as the marker attributes for the construction of predictive models. These selected peaks, such as bins 2410–2419, 2450–2459 and 6590–6599 Da, have indicated remarkable differences between MRSA and MSSA, which were effective in the prediction of MRSA. The independent testing has revealed that the random forest model can provide a promising prediction with the area under the receiver operating characteristic curve (AUC) at 0.8450. When comparing to previous works conducted with hundreds of MS spectra, the proposed scheme demonstrates that incorporating machine learning method with a large-scale dataset of clinical MS spectra may be a feasible means for clinical physicians on the administration of correct antibiotics in shorter turn-around-time, which could reduce mortality, avoid drug resistance and shorten length of stay in hospital in the future.

## Introduction

Methicillin-resistant *Staphylococcus aureus* (MRSA) is a superbug that is associated with resistance to most antibiotics, increased morbidity, and mortality [1, 2]. Successful treatment of MRSA depends largely on the rapid and correct administration of glycopeptide antibiotics, such as vancomycin or teicoplanin [2]. Administration of glycopeptides is usually guided by an antibiotic susceptibility testing (AST) [2, 3]. However, the AST-guided administration of correct antibiotics can cause considerable delay in using glycopeptide because the AST is reported days after specimen collection [2]. Paper disc, micro-broth dilution, macro-broth dilution or agar dilution are common AST methods in the current clinical microbiology laboratory. However, nucleic acid testing of the mecA gene by polymerase chain reaction (PCR) can detect the presence of the MRSA culprit gene [4]. All of the test methods are accurate; however, the methods share some common disadvantageous characteristics, including high cost, labor intensity and long turn-around-time [5].

Matrix-assisted laser desorption ionization-time of flight mass spectrometry (MALDI-TOF MS) is a potential tool to address the unmet need of detecting MRSA rapidly and accurately. MALDI-TOF MS is currently a widely used method for bacterial species identification in clinical microbiology laboratories [6–10]. MALDI-TOF MS measures whole bacterial cells and generates massive amounts of protein expression patterns [11]. Bacterial species can be identified by comparing ribosomal protein patterns with a reference database [12, 13]. Comparing to the conventional process of phenotypic and biochemical testing, the MALDI-TOF MS is a rapid, precise and cost-effective method for the identification of bacterial species.

**Figure 1:**
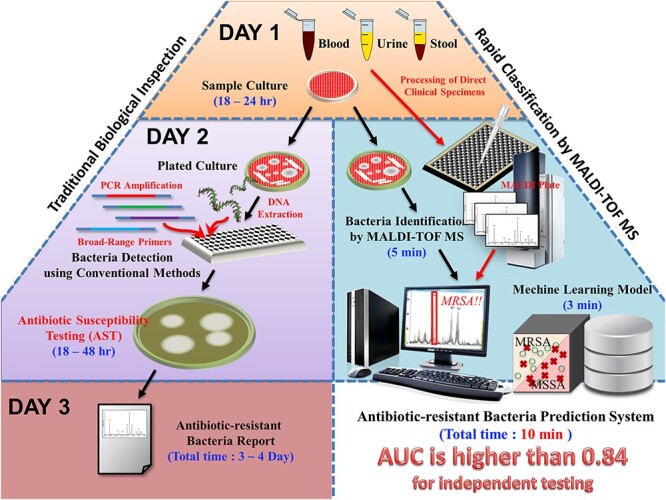
Comparison of processing workflows between traditional and proposed schemes. In clinical microbiology laboratory, the MALDI-TOF MS is routinely used for identification of bacterial species when a bacterial colony is recovered from specimen. Thus, a MS spectrum would be generated in the process of bacterial species identification. Based on the MS spectrum generated in the existing workflow, three steps are necessary for a medical staff to use the proposed scheme. The first step is inputting the MS spectrum into the ML model. A predicted result then would be provided by the proposed ML model. Second, on the basis of the predicted result, together with the patient’s clinical conditions, a clinical microbiologist can provide the preliminary result of oxacillin susceptibility for *S. aureus*. Third, the caring physicians can adjust the management (e.g. different antibiotic, different drug administrating dose/frequency) for *S. aureus*-related infection by the preliminary result of oxacillin susceptibility provided by the proposed ML model. Generally, the preliminary result of oxacillin susceptibility could be provided around one day in advance to the final oxacillin susceptibility test. The faster susceptibility information would enable a guide more appropriate clinical management.

Predicting MRSA using MALDI-TOF MS is not a novel approach in biology [14, 15]. Most of the studies focus only on the relationship between single proteins and MRSA. These studies concluded that the expression of ribosomal proteins was different among MRSA and methicillin-susceptible *Staphylococcus aureus* (MSSA) isolates. However, the performance of a single protein for detecting MRSA is not robust enough and has not been widely validated for clinical application. The insufficient performance may be attributed to the limited information of single proteins. Applying computer science to analyze massive MALDI-TOF MS data could provide comprehensive insight into the differences in protein fingerprints between MRSA and MSSA isolates. Figure 1 presents a comparison of processing workflows between the conventionally experimental process and the newly proposed MS-based scheme.

This study designed a new scheme to classify MRSA and MSSA isolates by detecting their important features from MALDI-TOF MS data. Using data mining and machine learning (ML) methods, the ions of the whole mass-to-charge (m/z) range from 2000 to 20 000 Dalton (Da) were investigated and thoroughly and comprehensively evaluated. All features with obvious differences between MRSA and MSSA isolates were selected and analyzed to construct models. With these models, physicians could be notified of the risk of MRSA infection rapidly and accurately. Moreover, they could also identify the essential features contributing to the classification results. These models could have an impact on the clinical management of patients with infectious diseases.

## Materials and Methods

**Figure 2:**
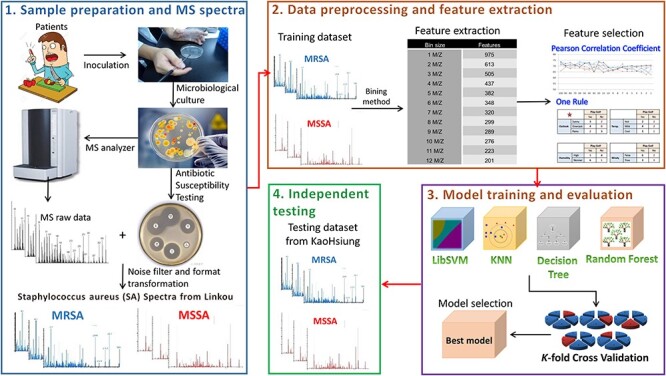
Analytical flowchart of this study. There are four steps: (1) sample preparation and MS spectra, (2) data preprocessing and feature extraction, (3) model training and evaluation and (4) independent testing.

The analytical flowchart for this study is depicted in Figure 2, consisting of (1) sample preparation and MS spectra, (2) data preprocessing and feature extraction, (3) model training and evaluation and (4) independent testing. First, bacterial samples were obtained from patients and cultured in the Linkou Chang Gung Memorial Hospital (CGMH). The cultured bacterial samples were subjected to MALDI-TOF MS to obtain the MS spectra with the annotation of MRSA or MSSA, as analyzed by antibiotic susceptibility testing (AST). Before the construction of predictive models, two different measures, the Pearson correlation coefficient (PCC) and one rule (OneR) attribute evaluation, were adopted to rank all features (e.g. spectra peaks) according to their discriminating abilities. Next, the ranked peaks were sequentially forward selected to train the predictive model. Then, the predictive models trained using different feature sets were evaluated for their performance in classifying MRSA and MSSA isolates based on k-fold cross-validation. Finally, the independent testing dataset, collected from Kaohsiung branch of CGMH, was used to test the final model with the best cross-validation performance. The detailed process of each step is described as follows.

### Sample preparation and MS spectra

Clinical specimens were continuously collected daily from various wards of both the Linkou and Kaohsiung branches in 2016 and delivered to the CGMH clinical microbiology laboratory. The specimen types included blood, respiratory tract specimen (i.e. sputum, bronchial wash and bronchoalveolar lavage), sterile cavity fluid (i.e. ascites, dialysates, pleural effusion, pericardial effusion, cerebrospinal fluid and synovial fluid), tip of implant, urine, wound and others. Blood specimens were collected after aseptic preparation, and cultured in trypticase soy broth (Becton Dickinson, MD, USA). Positive culture results were detected using an automated detection system (BD BACTEC™ FX; Becton Dickinson, MD, USA). Blood was drawn from positive blood culture bottles and spread onto blood plate (BP) agar for subculture (Becton Dickinson, MD, USA). Sputum specimens with acceptable quality [16] were used. Respiratory specimens were inoculated onto BP agar (Becton Dickinson, MD, USA), eosin methylene blue (EMB) agar (Becton Dickinson, MD, USA), Columbia naladixic acid (CNA) agar (Becton Dickinson, MD, USA) and chocolate agar (Becton Dickinson, MD, USA). Specimens obtained from sterile cavity fluid were inoculated on BP, EMB, CNA, chocolate agar and thioglycollate broth (Becton Dickinson, MD, USA). When positive growth was noted in thioglycollate broth, subculture on BP agar was conducted. A semi-quantitative culture method described by Maki *et al*. [17] was used for testing the tip of implants. Urine specimens were inoculated by a quantitative loop on BP and EMB agar. For specimens collected from wounds, 1.2 ml 0.9% saline was used for rinsing when the specimens were obtained by swab. The rinsed saline was inoculated on BP, EMB, CNA and chocolate agar. For pus collected from wounds, the specimens were directly dropped onto the agar and into thioglycollate broth. The agar and broth were incubated in a 37°C CO_2_ incubator for 18–24 h. Single colonies grown on agar plates were selected for further analysis. *S. aureus* was identified based on colony morphology, a coagulase test and MALDI-TOF MS (Bruker Daltonics GmbH, Bremen, Germany). The paper disc method using cefoxitin was performed to discriminate MRSA from MSSA, based on the Clinical & Laboratory Standards Institute guidelines.

All MS spectra were generated during routine tests in the clinical microbiology laboratory. Thus, we analyzed the unique bacterial isolates only once; no technical or biological replications were performed. Analytical measurements of MALDI-TOF MS were conducted according to the manufacturer’s instructions (Bruker Daltonics GmbH, Bremen, Germany). Single colonies grown on agar were picked and smeared to thin films on a MALDI steel target plate. One microliter of 70% formic acid was applied to the films and they were dried at room temperature (25°C). One microliter matrix solution (50% acetonitrite containing 1% α-cyano-4-hydroxycinnamic acid and 2.5% trifluoroacetic acid) was then added to the films. The sample matrix was dried at room temperature before analysis by MS. MALDI-TOF was conducted on a Microflex LT mass spectrometer (Bruker Daltonics GmbH, Bremen, Germany). Mass spectra were obtained under the following settings: linear positive mode, accelerating voltage: +20 kV and nitrogen laser frequency: 60 Hz. A total of 240 laser shots on each sample spot were used for measurement. The Bruker Daltonics Bacterial Test Standard was used for external calibration of the spectra. Flexanalysis 3.4 (Bruker Daltonics GmbH, Bremen, Germany) was used for spectral processing. The Savitzky–Golay algorithm was set for spectral smoothing. The spectral baseline was subtracted using the top hat method. The signal-to-noise ratio threshold was set as 2. *S. aureus* was determined by Biotyper 3.1 (Bruker Daltonics GmbH, Bremen, Germany) on the basis of the processed spectra. All spectra of the cases reached acceptable quality (log score ≥ 2, defined by the manufacturer’s instruction). Spectra ranging from 2000 to 20 000 Da were collected for further analysis.

### Data preprocessing and feature extraction

In the MALDI-TOF MS spectra, the peaks, which were the m/z values with sufficient intensity, were extracted and regarded as fingerprint signatures for the construction of predictive models. In an initial scanning through all MS spectra, due to the isotope of various atoms, it was noticed that many peaks with a little difference in m/z values might be referred to as the same peptide species. To deal with the shifting problem of peaks among different spectrums, a binning method was adopted to group the large-scale peaks, which ranged from 2000 to 20 000 Da, into a smaller number of ‘bins.’ Supplementary Figure S1 presents a schematic diagram of the binning method used in this work. Given two spectra marked in different colors (red and black), the peaks located within the same bin were considered to be the same attribute. In the binning method, various values of bin size, ranging from 1 to 15 Da, were tested to obtain the best performance in discriminating between MRSA and MSSA MS spectra. Note that ‘2410–2419’ was equivalent to [2410, 2420) in this study to represent a peak with adequate intensity in the interval that included the lower bound but did not include the upper bound.

In this study, two different data types, categorical and numerical values were considered for the representation of the peaks. For the categorical data type, the values 1 and 0 indicated the presence and absence of a peak, respectively, if its intensity is higher than a specified cutoff value. For the numerical data type, in each MS spectra, all the intensity values were normalized by their mean value and the standard deviation. Namely, the normalized intensity value of each peak was regarded as the training attribute for model construction. However, in a mass peptide fingerprint, the intensity values of these peptides might be affected by various factors, including the amount of bacteria loaded onto the steel plate, degree of cell lysis and other manual processes used to prepare the sample-matrix mixture prior to the analysis. Even if two spectrums were obtained from the same clinical isolate, the distribution of peaks, as well as their intensity values in the two spectrums might be different. Therefore, the intensity values of all identified peptides were normalized for each spectrum. Herein, the z-score normalization method was applied to each spectrum with an attempt to avoid extreme concentration values. The normalizing function for the peak value *x_ij_* of spectrum *i* is defined as:

$${z}_{ij}=\frac{x_{ij}-{\mu}_i}{\sigma_i}$$

where *μ_i_* is the mean of all intensity values and *σ_i_* is the standard deviation of spectrum *i* [18]. After z-score normalization, the distribution of all *z* values will be close to a normal distribution.

### Feature selection and construction of ML models

In general, we can adopt all attributes (bins) as the training features to construct a predictive model for classification between MRSA and MSSA samples. Due to the potential noise peaks included in the MS spectra, sometimes, the consideration of all attributes might induce a lower discriminating power. Therefore, sequential forward selection (SFS) [19, 20], an incremental strategy for selecting the final attribute set, was utilized to determine the final composition of attributes in an attempt to determine the informative attributes to differentiate MRSA from MSSA samples. SFS was applied on two different feature sets, which were selected using the PCC and OneR, to yield better predictive performance.

PCC is a metric used to detect the linear dependence (correlation) between two variables *X* and *Y* by generating a value between −1 and 1:

$$\rho \left(X,Y\right)=\frac{\operatorname{cov}\left(X,Y\right)}{\sigma_X{\sigma}_Y}=\frac{E\left[\left(X-{\mu}_X\right)\left(Y-{\mu}_Y\right)\right]}{\sigma_X{\sigma}_Y}$$

where cov(*X*,*Y*) is the covariance between *X* and *Y*, and *σ_x_* (*σ_y_*) is the standard deviation of *X* (*Y*). As presented in Supplementary Figure S2, all positive (MRSA) and negative (MSSA) samples of the training dataset are labeled as +1 and −1, respectively. To calculate the PCC value for a given attribute (peak), the samples with/without this attribute were labeled as 1/0. In the investigation of the correlation between a given attribute and the sample distribution, a higher PCC value indicated that the evaluated attribute had a higher correlation with the distribution of positive and negative samples.

OneR is a rule-based strategy to evaluate the classifying ability of each attribute [21]. In this investigation, each attribute was regarded as a single rule for classifying MRSA and MSSA samples. OneR was used as a one-level decision tree (DT) to generate a set of rules that tested one particular attribute [22]. There were three main steps in the OneR investigation of each attribute:

(i) Two branches for the attribute values (1 and 0).
(ii) Each branch was assigned a class label (MRSA or MSSA) with the highest frequency.
(iii) Calculation of error rate for each branch: proportion of samples that did not belong to the assigned class of their corresponding branch.

After calculating the error rate against all attributes, all of them were ranked according to the error rate in ascending order. The attribute containing the lowest error rate represented the best classifying ability. Supplementary Figure S3 provides an example of the OneR evaluation of three attributes.

In this study, four ML models, DT, random forest (RF), K-nearest neighbor (KNN) and support vector machine (SVM), were adopted. The ‘rpart’ (version 4.1.15) [23], ‘ranger’ (version 0.12.1) [24], ‘kknn’ (version 1.3.1) [25] and ‘e1071’ (version 1.7.3) [26] packages of R software (version 3.6.3, R Foundation for Statistical Computing, https://www.r-project.org/) were utilized to generate the DT, RF, KNN and SVM models, respectively. Detailed descriptions of these methods are provided in the supplementary methods. For each bin size, the nested 5-fold cross-validation was performed to determine the optimal learning parameters of the four ML models with a grid search by the inner loop. Supplementary Table S1 shows descriptions of learning parameters used in the nested cross-validation for the four ML methods. The outer loop was used to rank the features and determine the best feature composition. Specifically, the features were ranked according to the PCC or OneR evaluations in each training fold. Then, the ranked peaks were sequentially forward selected to train the predictive model and evaluate its predictive performance in the outer loop. Consequently, the optimal parameters and final feature set could be determined for each bin size.

### Performance measurement of predictive models

The MRSA prediction models trained using various ML methods were evaluated via 5-fold cross-validation. When the optimal parameters and final feature set were determined, 5-fold cross-validation was then employed to evaluate their predictive performance based on the training data. In the 5-fold cross-validation, all the positive (MRSA) and negative (MSSA) training samples were divided into five subgroups with approximately equal data sizes. The ratio of the validation dataset to the training dataset was 1:4, and the cross-validation process was repeated five times with an attempt to regard each subgroup as the validation dataset once. After a round of 5-fold cross-validation, the results of five validations were combined to generate a single estimation. To estimate the predictive performance of each trained model, metrics, such as sensitivity (SEN), specificity (SPE), accuracy (ACC) and the Matthews correlation coefficient (MCC) were utilized:

$$\mathrm{SEN}=\frac{\mathrm{TP}}{\mathrm{TP}+\mathrm{FN}}$$

$$\mathrm{SPE}=\frac{\mathrm{TN}}{\mathrm{FP}+\mathrm{TN}}$$

$$\mathrm{ACC}=\frac{\mathrm{TP}+\mathrm{TN}}{\mathrm{TP}+\mathrm{TN}+\mathrm{FP}+\mathrm{FN}}$$

$$\mathrm{MCC}=\frac{\left(T\mathrm{P}\times \mathrm{TN}\right)-\left(\mathrm{FN}\times \mathrm{FP}\right)}{\sqrt{\left(\mathrm{TP}+\mathrm{FN}\right)\times \left(\mathrm{TN}+\mathrm{FP}\right)\times \left(\mathrm{TP}+\mathrm{FP}\right)\times \left(\mathrm{TN}+\mathrm{FN}\right)}},$$

where TP, TN, FP and FN denote the prediction of true positives, true negatives, false positives and false negatives, respectively. The SEN and SPE indicated the proportion of correct predictions for positive (MRSA) and negative (MSSA) samples, respectively. The ACC combined the results of true-positive and true-negative predictions. Because the size of the positive dataset was not balanced by the size of the negative dataset, the MCC metric could provide a reasonable evaluation for classifiers [22, 27]. In this investigation, the area under the receiver operating characteristic (ROC) curve (AUC) was considered as the primary metric for the performance comparison among different ML models [28]. It should be noted that the optimal parameters and feature set were determined based on the best AUC value. In order to ensure the robustness of independent testing, the standard deviation metric was further considered to determine the optimal bin size, ML parameters and the final feature set. Prior to the independent testing, the whole training dataset was used to construct a predictive model with the optimal bin size, ML parameters and feature set.

## Results

### Data statistics of MRSA and MSSA samples in training and independent testing datasets

The MALDI-TOF MS data obtained from the CGMH Linkou branch served as the training dataset. *S. aureus* was isolated from 3338 cases, including 1954 males and 1384 females. As presented in Table **1**, 1056 MRSA isolates and 898 MSSA isolates have been identified in the male population. For the female population, 712 MRSA isolates and 672 MSSA isolates were identified. In the training dataset, the average ages (standard deviation) of the male and female individuals were 54.68 (25.46) and 52.90 (24.16) for MRSA and MSSA, respectively. According to the AST results, the ratio of MRSA (1768 instances) against MSSA (1570 instances) samples was approximately 1:1. Additionally, MRSA specimen type distribution revealed that the wound specimen was the most predominant specimen type, followed by the respiratory tract and blood specimens; in MSSA, the top three most common specimen types were the same as those of MRSA, namely wound, blood and respiratory tract specimens.

**Table 1:** Data statistics of MRSA and MSSA samples in the training and testing datasets

	Training dataset		Testing dataset	
	MRSA	MSSA	MRSA	MSSA
Age	54.68 ± 25.46	52.90 ± 24.16	55.90 ± 22.65	57.44 ± 19.61
Gender				
Male	1056	898	448	474
Female	712	672	284	314
Specimen type				
Blood	171	184	239	310
Respiratory tract	542	290	52	33
Sterile body fluid	51	43	17	29
Uninary tract	86	135	26	33
Wound	825	773	365	316
Others	93	145	33	67
Total	1768	1570	732	788

In this investigation, the MALDI-TOF MS spectra obtained from the CGMH Kaohsiung branch was regarded as the independent testing dataset. *S. aureus* was isolated from 1520 cases, including 922 men and 598 women. As presented in Table 1, 448 MRSA isolates and 474 MSSA isolates have been identified in the male population. In the female population, 284 MRSA isolates and 314 MSSA isolates were identified. The average ages (standard deviation) of male and female individuals were 55.90 (22.65) and 57.44 (19.61) for MRSA and MSSA, respectively. The ratio of MRSA to MSSA was 732:788, which was also approximately 1:1, in the testing dataset. The composition of the specimens in the testing dataset was similar to that of the training dataset, where the top three major specimen types were wound, blood and respiratory tract specimens for both MRSA and MSSA isolates. The independent testing dataset was adopted to evaluate the optimal model, which was trained by using the entire training dataset with the optimal ML parameters and feature set.

### Comparisons of MS spectra between MRSA and MSSA

**Figure 3:**
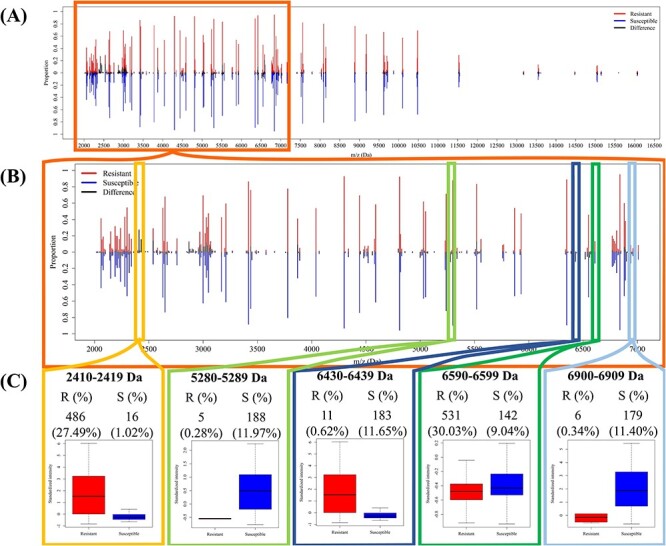
Comparison of peak distributions between MRSA and MSSA spectra. (**A**) Distribution of peaks in the region between 2000 and 17 000 Da. (**B**) Distribution of peaks in the region between 2000 and 7000 Da. (**C**) The intensity distribution and number (proportion) of spectra with specific peaks.

Figure 3 demonstrates the peak distribution in the MS spectra to investigate the differences between MRSA and MSSA isolates. The number of isolates with adequate intensity at specific peaks is shown in Figure 3A. It should be noted that the number of isolates with adequate intensity at specific peaks was independent of their intensities. Figure 3A provides an overview of the peak distribution over a wide range, starting from 2000 to 17 000 Da. Since the number of peaks occurring within 7000–17 000 Da is much less than that occurring within 2000–7000 Da, a detailed view of the peak distribution ranging from 2000 to 7000 Da is displayed in Figure 3B. In other words, peaks with m/z values lower than 7000 Da would be more informative for further investigation. Figure 3A implies that the majority of peaks of approximately 3000–5000 Da in both MRSA and MSSA isolates show adequate intensities. Figure 3C further illustrates the distribution of intensity and number (proportion) of spectra with adequate intensity at the specific peak. These results indicated that the peaks shown in the range lower than 3000 Da had larger intensities. In contrast, the peaks in the range of 3000–5000 Da usually had lower intensities. Additionally, the peak distribution in the range between 2000 and 3000 Da, shown in Figure 3, illustrates the differences between MRSA and MSSA isolates. Note that the region with blank indicates the difference between MRSA and MSSA spectra. Compared with MSSA, MRSA spectra tended to show peaks in the range of 2410–2419 and 6430–6439 Da. Therefore, these specific regions were thought to be highly related to the classification of MRSA and MSSA isolates, and were expected to be selected by feature selection methods.

### Feature evaluations for prediction

In this study, ribosomal proteins, which correspond to specific M/Z values of a spectrum, were used to construct the classification models. The M/Z values of the ribosomal proteins, between 2000 and 20 000 Da were detected by MALDI-TOF MS. This large range was split into different bin sizes to extract applicable features and further deal with the peak shifting problem. There were 15 different bin sizes used in this study: 1–15 Da. For example, if the bin size was 10 Da, the first bin ranged from 2000.000 to 2009.999 Da, which was denoted as ‘2000–2009.’ For instance, if a spectrum had peaks at 2003 or 2008 Da, the value of this feature was 1, indicating that this isolate had peaks in this range. In addition, the features were removed if the number of spectra showing the peak in the range was lower than 5% of the number of spectra. Table 2 shows the number of features without the feature-selection strategy for different bin sizes. Basically, the number of features decreased as the bin size increased. Table 2 also provides the number of features, which are sequentially forward selected, based on the ranking of PCC and OneR for different bin sizes when the RF model was adopted. In this investigation, PCC and OneR provided two different feature sets ranked according to their relevance in discriminating between MRSA and MSSA isolates. In addition, Supplementary Table S2 provides a table of the top 20 features selected by PCC for a bin size of 10 Da. The features ranked with 1st, 2nd, 15th and 20th covered the m/z values approximately 2400 Da, which was regarded as the preferable peaks for MRSA samples. These results are also consistent with those in Figure 3. Particularly, the range between 2410 and 2419 Da yielded the highest score among all features.

**Table 2:** Number of features with or without feature selection for different bin sizes. Given a specific bin size (in the first column), without the feature selection method, the number of features (peaks) containing adequate intensity value is provided in the second column. In this investigation, the peaks that occur in less than 5% of the samples are not considered in the feature selection process. The number of sequentially forward selected features (peaks) based on the ranking of PCC and OneR are provided in the third and fourth columns, respectively

Bin size (M/Z)	Number of features	Number of selected features (PCC)	Number of selected features (OneR)
1	936	501	706
2	662	237	526
3	521	343	508
4	455	375	447
5	391	336	296
6	349	243	338
7	327	281	323
8	299	216	281
9	284	245	264
10	268	193	201
11	259	222	239
12	245	178	238
13	243	227	221
14	223	220	202
15	219	201	219

### Comparisons of the predictive models

**Figure 4:**
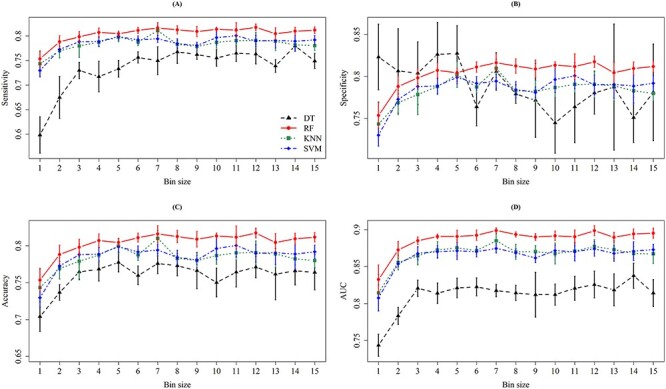
The means and standard deviations of (**A**) sensitivity, (**B**) specificity, (**C**) accuracy and (**D**) AUC on 5-fold cross-validation for different bin sizes when the intensity is not incorporated. In each column of the four subfigures, the means and standard deviations are represented by dots and bars, respectively, for the four ML methods, namely DT (black), RF (red), KNN (green) and SVM (blue).

**Figure 5:**
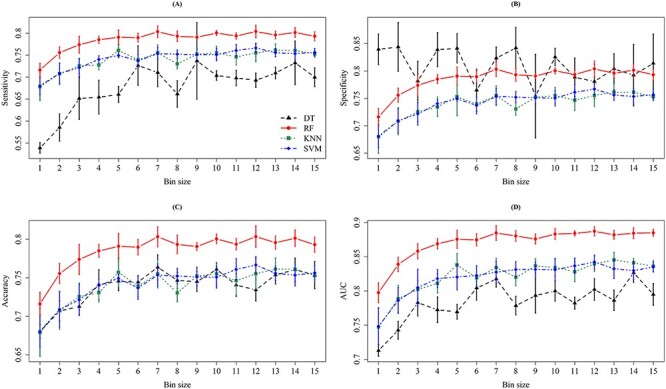
The means and standard deviations of (**A**) sensitivity, (**B**) specificity, (**C**) accuracy and (**D**) AUC on five cross-validation for different bin sizes when the intensity is incorporated. In each column of the four subfigures, the means and standard deviations are represented by dots and bars, respectively, for the four ML methods, namely DT (black), RF (red), KNN (green), and SVM (blue).

In this study, we developed different classification models using different classifiers and feature selection strategies. Specifically, 4 ML methods, two feature sets determined by PCC and OneR, and 15 bin sizes were taken into consideration. Figures 4 and 5 show the results for different bin sizes without and with the consideration of intensity values, respectively. Nested cross-validation was adopted to determine the optimal parameters of ML methods and the final feature set. When considering the PCC-based feature set, the models with bin sizes of 7, 10 or 12 Da achieved better ACC and AUC values. More importantly, the model trained using bin size 10 Da attained the lowest standard deviation, which indicated a more stable prediction performance. Supplementary Table S3 shows the top 16 predictive models with their detailed information, including classifier, bin size, feature-ranking method, SEN, SPE, ACC, MCC and AUC.

As shown in Supplementary Table S3, the RF-based model trained with bin size 7 Da and PCC-ranked feature set can provide the best AUC (0.8997) in MRSA prediction. However, the ACC fluctuation (standard deviation) tends to be higher (0.0074). To ensure the robustness of the model, a lower standard deviation was the major concern. As a result, the RF model trained using the PCC feature set with the bin size 10 Da was selected. Since intensity could represent the signal strength at a specific region, we further took it into consideration using a bin size of 10 Da to construct the classification models. Supplementary Table S4 demonstrates the performance of different classifiers when considering the intensity value and a bin size of 10 Da. Note that the intensity values were normalized by their mean and standard deviation. The AUC value of the RF-based model trained with PCC feature set (0.8853) in Supplementary Table S4 is slightly lower than that (0.8921) in Supplementary Table S3. Without considering the intensity values of the peaks, the RF-based model could provide better ACC (0.8101) and AUC (0.8921) values. The AUC slightly decreased when the RF-based model was trained using the normalized intensity values, which implied that the feature of normalized intensity values could improve the classification between MRSA and MSSA samples. Nevertheless, the presence or absence of peaks was sufficiently useful in the prediction of MRSA samples.

### Investigation of informative peaks

Table 3 shows that the optimal model was developed by DT and PCC feature selection when the bin size was 10 Da. We then further investigated the summary statistics for these selected features. Specifically, there were 108 features selected by the PCC, and 110 features selected by OneR. Table 4 demonstrates the information about the peaks selected by these two feature selection methods simultaneously. Some informative peaks have been mentioned in previous studies that are shown in the last column of Table 4. However, we found informative peaks that had not been mentioned so far. Specifically, most of the selected peaks showed significant differences between MRSA and MSSA isolates. However, previous studies have mainly found that MRSA has specific peaks. Specifically, 2410–2419 Da [15, 29], 6590–6599 Da [30], 2450–2459 Da [29, 31, 32], 5000–5009 Da [15, 30], 2540–2549 Da [29, 31], 6420–6429 Da [30], 5520–5529 Da [15, 30], 3030–3039 Da [30] and 3890–3899 Da [29, 30] have been reported. We also found that the number of MRSA peaks was larger than that of MSSA peaks in the range of 2430–2439 Da, which had not been mentioned before. In contrast, few studies mentioned the size of the MSSA peaks. The proportion of peaks between 5280 and 5289 Da for MSSA spectra was 11.97% higher than that of the MRSA spectra. In addition, the range of 6900–6909 Da also indicated that the MSSA isolates had more peaks than MRSA did. Even though we needed 108 features to construct an optimal classifier to discriminate between MRSA and MSSA isolates, these data statistics could also provide information for further experiments to identify the corresponding peptides.

**Table 3:** Prediction performance when bin size is 10 in the independent testing

Classifier	#Features	SEN	SPE	ACC	MCC	AUC
DT	268	0.6981	0.7284	0.7138	0.4267	0.7497
	47	0.6981	0.7284	0.7138	0.4267	0.7497
RF	268	0.7650	0.7652	0.7651	0.5300	0.8486
	193	0.7664	0.7665	0.7664	0.5326	0.8450
KNN	268	0.6981	0.698	0.6980	0.3958	0.7672
	46	0.7527	0.7500	0.7513	0.5025	0.8289
SVM	268	0.7418	0.7424	0.7421	0.4839	0.8110
	209	0.7418	0.7424	0.7421	0.4839	0.8097

**Table 4:** Comparison of the number of samples containing the selected peaks between MRSA and MSSA

Peak range	#MRSA (%)	#MSSA (%)	Difference (%)	PCC rank	OneR rank	Reference
2410–2419	486 (27.49)	16 (1.02)	470 (26.47)	1	1	[15, 29]
2430–2439	275 (15.55)	1 (0.06)	274 (15.49)	2	17	
6590–6599	531 (30.03)	142 (9.04)	389 (20.99)	3	2	[30]
5280–5289	5 (0.28)	188 (11.97)	−183 (−11.69)	4	3	
2450–2459	224 (12.67)	2 (0.13)	222 (12.54)	5	40	[29, 31, 32]
6900–6909	6 (0.34)	179 (11.40)	−173 (−11.06)	6	5	
6430–6439	11 (0.62)	183 (11.66)	−172 (−11.03)	7	6	
6520–6529	0 (0)	136 (8.66)	−136 (−8.66)	8	9	
3450–3459	10 (0.57)	153 (9.75)	−143 (−9.18)	10	7	
10 460–10 469	10 (0.57)	138 (8.79)	−128 (−8.22)	11	10	
5000–5009	1 (0.06)	105 (6.69)	−104 (−6.63)	12	11	[15, 30]
6560–6569	6 (0.34)	102 (6.50)	−96 (−6.16)	13	12	
2540–2549	512 (28.96)	232 (14.78)	280 (14.18)	14	14	[29, 31]
6420–6429	1658 (93.78)	1317 (83.89)	341 (9.89)	16	8	[30]
5520–5529	1472 (83.26)	1098 (69.94)	374 (13.32)	17	4	[15, 30]
11 550–11 559	4 (0.23)	81 (5.16)	−77 (−4.93)	18	16	
7030–7039	2 (0.11)	66 (4.20)	−64 (−4.09)	19	21	
2220–2229	42 (2.38)	122 (7.77)	−80 (−5.4)	22	15	
3030–3039	751 (42.48)	487 (31.02)	264 (11.46)	24	20	[30]
3890–3899	84 (4.75)	160 (10.19)	−76 (−5.44)	30	18	[29, 30]

Box plots were adopted to investigate the difference in intensities between MRSA and MSSA peaks, and are shown in Figure 3C. The remarkable difference observed from the box plots implied that these peaks were able to discriminate between MRSA and MSSA isolates. Although some box plots did not show remarkable differences, the number of peaks appearing in the specific region could help classify MRSA and MSSA isolates. In addition to the individual peaks, we also investigated the pairwise correlation between any pair of two peaks. Supplementary Figure S4 illustrates the heatmap of PCC between two selected peaks in MRSA and MSSA samples. This investigation implied that the correlations of selected peaks for MRSA samples tended to be higher than those for MSSA samples. For instance, the correlation between peaks 2410–2419 Da and 3000–3009 Da in MRSA samples is higher than that in MSSA samples. In addition, the PCCs between the peak 2410–2419 Da and other selected peaks in MRSA and MSSA samples are provided in Supplementary Table S5. The number of samples containing both peaks (the peak 2410–2419 Da and another specific peak) with or without adequate intensity values are provided for MRSA and MSSA samples. Then, the PCC value was calculated for each pair of two selected peaks. For instance, the number of samples containing both peaks 2410–2419 Da and 3000–3019 Da with adequate intensities in MRSA (469 samples) is higher than that in MSSA (six samples). Additionally, the absolute values of PCCs in MRSA samples are higher than those in MSSA samples except the pair containing peak 6420–6429 Da.

### Performance of independent testing

**Figure 6:**
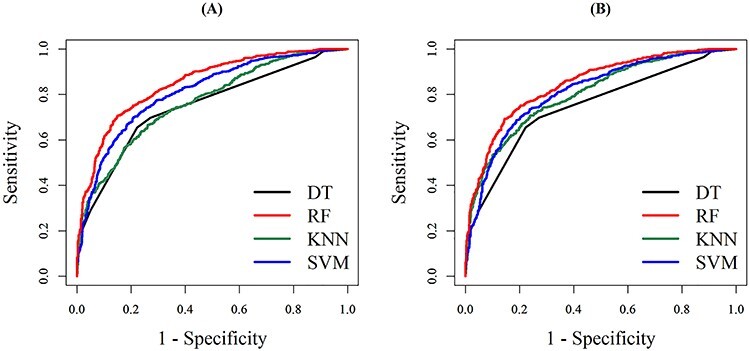
The ROC curve for independent testing when the bin size is 10 Da. (**A**) and (**B**) are the ROC curves for the four ML models trained with and without, respectively, the consideration of feature-selection strategy.

Table 3 shows the predictive performances of the four ML methods trained with a bin size of 10 Da using the independent testing data. This investigation demonstrated that the models trained using the selected features could provide a better predictive power, especially for the KNN model in which the AUC value increased from 0.7672 to 0.8289. In the independent testing, the RF model performed best with SEN, SPE, ACC, MCC and AUC at 0.7664, 0.7665, 0.7664, 0.5326 and 0.8450, respectively. The testing results indicated that the consideration of fewer, but more informative features, could provide better discriminating power. In addition, the ROC curves of the four models with and without the feature selection strategy are shown in Figure 6.

## Discussion and Conclusion

Predicting MRSA using MALDI-TOF MS is not a novel approach in biology, but the reported performance varies considerably between studies. Previous studies have attempted to associate a few single peptides/proteins to MRSA isolates [14, 15, 33]. Alksne *et al.* had used 2412 ± 2 Da as the indicator peak to identify MRSA, which attained the sensitivity of 61% and specificity of 100% on 272 *S. aureus* isolates [33]. If we adopted the same criterion to identify MRSA isolates, Table 4 has revealed that only 27.49% MRSA samples contain the peaks with adequate intensity in the range of 2412 ± 2 Da. In recent years, several studies [34–37] had adapted ML algorithms to conduct the detection of MRSA based on hundreds of MS spectra. Bai *et al.* [35] had adopted a two-step binning approach with genetic algorithm to construct SVM classifier based on 727 *S. aureus* isolates, and the predictive accuracy was around 72% in average. Sogawa *et al.* [37] also applied the SVM approach on 160 *S. aureus* isolates, in which 100 samples were used to train the model and the remaining 60 samples were regarded as testing data. The sensitivity and specificity values were 86.7 and 93.3%, respectively. Tang *et al.* [34] had examined 10 MRSA and 10 MSSA clinical isolates, and the prediction accuracy was higher than 90%. Kim *et al.* [36] had exploited the DT algorithm to train the predictive model using 320 *S. aureus* isolates; additionally, 181 *S. aureus* isolates were used to test the DT model, which attained the sensitivity and specificity at 87.6 and 71.4%, respectively.

Regarding sample size, the number of MRSA and MSSA isolates used in this study is much larger than those in previous reports [14, 15, 33–35]. With a larger sample size, more bacterial diversity is included and the possibility of overfitting can be mitigated. Moreover, ML models were trained using Linkou CGMH data and the ML models were independently tested using the Kaohsiung CGMH data. External validation was the optimal validation method to examine the robustness of ML models; however, external validation on a large-scale was not done in other studies. A possible bias and lack of robustness could be the reasons why the reported results have not been widely applied in clinical practice. Regarding the technical aspects of MALDI-TOF analysis, we did not use technical replicates or biological replicates, which can be used to increase the sample size and improve reproducibility [34]. In the routine workflow of MALDI-TOF analysis in clinical microbiology laboratories, only single MALDI-TOF measurements were available for a bacterial isolate. With the aim of developing an ML tool useful in clinical practice, we used single MALDI-TOF measurements alone to train, validate and independently test ML models. For reproducibility, which could be compromised using single MALDI-TOF measurements, we proposed and validated a binning method that could deal with the problem of peak drift/shift issues. In brief, we developed and validated an MRSA prediction model using a comprehensive dataset with a rigorous validation process, which has not yet been reported in previous studies. Additionally, the proposed scheme is ready to be used in a clinical setting because the ML model was trained and validated using data consecutively collected from clinical daily work in the real world.

In this study, all the MS data were generated during routine tests in the clinical microbiology laboratory. Thus, we analyzed unique bacterial isolates only once; no technical or biological replications were performed. We developed an applicable tool for fast detection of MRSA isolates in clinical practice. In the routine work of clinical microbiology laboratories, we usually spread bacteria onto a single spot of the analytical plate and have only a single analytical measurement of MALDI-TOF MS spectra for each bacterial isolate. For real integration and application into real-world workflow, we used single measurements without replications and evaluated whether MRSA detection was possible using the proposed methods. The major obstacle in MRSA detection based on MALDI-TOF MS spectra is the peak-shifting issue of MALDI-TOF MS [11, 38]. Consequently, we proposed and evaluated if the binning method was an adequate method for preprocessing MALDI-TOF MS spectra to facilitate the incorporation of ML algorithms.

The reproducibility of the spectra is recognized as an intrinsic limitation of MALDI-TOF MS. Several studies have indicated the lack of reproducibility of the mass spectra when MALDI-TOF MS was used for strain typing or predicting AST. In fact, the peak-level reproducibility of peak presence/absence seems to be approximately 90%, as reported in a review article [39]. There are many factors which would affect the reproducibility of MS spectra. Regarding the media, in the study, we cultivated *S. aureus* isolates on BBL™ Trypticase™ Soy Agar with 5% Sheep Blood (TSA II) (Becton Dickinson, MD, USA) for 16–18 h to obtain single colonies for MALDI-TOF analysis. Direct on-plate preparation of the sample was adapted, in which the bacterial amount was only semi-quantitatively measured by experienced medical technologists. We conducted cell lysis and manual processes to prepare the sample-matrix mixture according to the manufacturer’s instructions (Bruker Daltonics GmbH, Bremen, Germany). As routinely processed in clinical microbiology laboratories, detailed information including bacterial amount, degree of cell lysis and other possible factors were not precisely defined. The score (log score ≥ 2) provided by Biotyper 3.1 (Bruker Daltonics GmbH, Bremen, Germany) could be the quality indicator showing that analytical processes were correctly performed. In the literature [40], in-tube extraction rather than on-plate extraction can provide more precise bacterial amounts, cell lysis, sample-matrix mixing and higher quality MS spectrum. However, given the aim of developing an ML tool useful in clinical practice, we should fit the ML tool into the existing workflow. In the current workflow of MALDI-TOF analysis, direct on-plate sample preparation is the only possible method for dealing with overwhelming numbers of test orders in clinical microbiology laboratories [41].

This investigation employed different feature selection methods, such as PCC and OneR to identify informative peaks for the construction of MRSA prediction models. Then four different classification algorithms were used to build the models. Evaluation by 5-fold cross-validation indicated that the selected peaks were effective in the prediction. According to PCC feature selection with a bin size 10 Da, 193 selected peaks could provide the best predictive power for the independent testing data (76.64% accuracy, 76.64% sensitivity, 76.65% specificity, 0.5326 MCC and 0.8450 AUC) based on the RF classifier. With rapid, accurate prediction, *S. aureus* will be classified immediately, which can help clinicians to administer appropriate treatment to patients without wasting any time.

There are limitations to this study. First, bacterial strains circulating in different regions vary considerably. Directly applying ML models to other areas or regions would be inappropriate, although we have validated the ML models with rigorous cross-validation and at an independent institute. In contrast, clinical microbiologists from other areas or regions could collect their own local MS data and follow the generalized approach proposed by the study to build a locally relevant ML model. Second, we trained and validated the ML models using data derived from the MALDI-TOF MS of Bruker Daltonics GmbH. MALDI-TOF MS spectra analyzed using other MS platforms may be different from Bruker systems. We did not conduct a comparison between different MALDI-TOF MS platforms in the study. Third, the informative peaks were not identified. Most of the informative peaks selected for feature selection presented a higher frequency in the resistant strains. Identification of the informative peaks would provide a more comprehensive view on the mechanism of antibiotic resistance and would be valuable for the development of novel antibiotics.

**Key Points**

- A binning method was incorporated to cluster MS shifting ions into a set of representative peaks based on a large-scale MS dataset of clinical *S. aureus*, including 2500 MRSA and 2358 MSSA isolates.
- In the evaluation of independent testing, the random-forest model performed best with the AUC at 0.8450, sensitivity at 0.7664 specificity at 0.7665 accuracy at 0.7664 and Mathews correlation coefficient at 0.5326.
- This work has demonstrated that incorporating machine learning method with a large-scale dataset of clinical MS spectra may be a feasible means for clinical physicians on the administration of correct antibiotics in shorter turn-around-time, which could reduce mortality, avoid drug resistance, and shorten length of stay in hospital in the future.

## Supplementary Material

Supplementary_bbaa138

## Notes

**Hsin-Yao Wang** is a medical doctor in the Department of Laboratory Medicine, Chang Gung Memorial Hospital at Linkou, Taoyuan City, Taiwan, and a student in the PhD. Program in Biomedical Engineering, Chang Gung University, Taoyuan City, Taiwan.

**Chia-Ru Chung** is a PhD candidate in the Department of Computer Science and Information Engineering, National Central University, Taoyuan City, Taiwan. Her research interests include bioinformatics, big data analytics, data mining and machine learning.

**Zhuo Wang** is a postdoc in Warshel Institute for Computational Biology, The Chinese University of Hong Kong, Shenzhen, China.

**Shangfu Li** is a postdoc in Warshel Institute for Computational Biology, The Chinese University of Hong Kong, Shenzhen, China.

**Bo-Yu Chu** received a master degree from the Department of Computer Science & Engineering, Yuan Ze University, Taoyuan City, Taiwan.

**Dr. Jang-Jih Lu** is currently the Director and Professor in the Department of Laboratory Medicine, Chang Gung Memorial Hospital at Linkou, Taoyuan City, Taiwan.

**Dr. Jorng-Tzong Horng** is now a Distinguished Professor in the Department of Computer Science and Information Engineering, National Central University, Taiwan, and a Professor in Department of Bioinformatics and Medical Engineering, Asia University, Taichung City, Taiwan. His research interests include database system, bioinformatics, computational biology, big data analytics, data mining and machine learning.

**Tzong-Yi Lee** is currently an associate professor at Warshel Institute for Computational Biology, School of Life and Health Sciences, The Chinese University of Hong Kong, Shenzhen, China. His research interests include bioinformatics, computational biology, systems biology, big data analytics, data mining and machine learning.
