# Supplementary material for: A large-scale investigation and identification of methicillin-resistant Staphylococcus aureus based on peaks binning of matrix-assisted laser desorption ionization-time of flight MS spectra
Source: Brief Bioinform. 2020 Jul 16;22(3):bbaa138. doi: 10.1093/bib/bbaa138 (PMC8138823; doi:10.1093/bib/bbaa138)
Supplement: Supplementary_bbaa138 [file supplementary_bbaa138.docx]

**Supplementary Information for ML Methods**

**Decision tree (DT)**

Given a training dataset *X* containing positive and negative samples, the entropy function of *X* is described as

$$Entropy(X)=-p^{+}\log_{2}p^{+}-p^{-}\log_{2}p^{-}$$

where *p*^＋^ is the percentage of positive sample and *p*^－^ is the percentage of negative samples. If the dataset contains *k* types pf classes or values, the entropy function should be revised as

$Entropy(X)=\sum_{i=1}^{k} -p^{i}\log_{2}p^{i}$.

The information gain can be then determined for measuring the purity of each attribute in a dataset and evaluating its effectiveness in sample classification. The information gain of an attribute *A* is defined as

$$Gain(X,A)=Entropy(X)-\sum_{w\in W(A)} \frac{|X_{w}|}{|X|}Entropy(X_{w})$$

where *W*(*A*) is the domain range of *A*, *X* is the sample set and *X_w_* is the sample set with attribute *A* equal to *w*. Then, all attributes (e.g. m/z peaks) were ranked by its information gain values. The attribute with the maximal information gain was selected as a root rule to start from the test of root node to a leaf node along an appropriate path. A tree-like structure was constructed for making decisions by testing the MRSA or MSSA samples in each node (including root node and internal nodes) till a leaf node. The path starting from root node to a leaf node will be concluded to a series of rules for making classification between MRSA and MSSA samples. A sequential coverage algorithm is typically applied to extract rules from DT based on greedy strategy [1]. In this investigation, the Rpart package in R was adopted to construct the classification model [2].

**Random forest (RF)**

Random forest (RF) is a sort of ensemble model that involves the aggregation of multiple decision tree classifiers. Based on the integration of multiple decision trees within a RF model, each tree was generated from a subset of *k* attributes randomly selecting from training dataset with a total of *m* attributes, where *k* is less than *m*. In this way, we can obtain multiple decision-making results. Typically, the majority voting method is adopted to integrate the results to make a final decision, based on the class label with the most votes. The performance of a RF decision-making system is associated with the dimension of random vector, which is the number of attributes (*k*) used in each decision tree. The value of *k* is typically defined as

$$k=\log_{2}m+1$$

where *m* is the total number of attributes in training dataset [3]. In this study, “randomForest” package which constructed in R [4], was utilized to construct RF classifiers based on various attribute sets.

***K*-Nearest Neighbors Algorithm (KNN)**

The nearest neighbor approach is an instance-based classifier used for determining the most similar instances, which were selected from all training data, to a given test instance, based on a distance function. Given a test instance, the most *k* similar instances are regarded as *k*-nearest neighbor algorithm (KNN) of the test data and the class assignment is determined in accordance with the proportion of KNN. Considering the training data and test data as the *n*-dimensional vectors in Gaussian space, the Euclidean distance function is usually applied to measure the distances between the test data and all training data. Given a test instance *t*, the Euclidean distance between *t* and a training instance *x* is defined as

$$d(t,x)=\sqrt{\sum_{k=1}^{n} (t_{k}-x_{k})^{2}}$$

where *n* is the size of attribute set. After finding out *k* nearest neighbors, the class labels of these *k* training instances might be inconsistent. A weighted distance voting method was used to carry out the class assignment for a test data. Class assignment *C*(*t*) of a test data *t* is determined by

$$C(t)=\mathop{\mathrm{argmax}}\limits_{v} \sum_{x_{i}\in KNNs} w_{i}I(x_{i}), \left\{\begin{matrix} I(x_{i})=1, if v=class label of x_{i} \\ I(x_{i})=0, if v\neq class label of x_{i} \end{matrix}\right.$$

where *v* is class label and *w_i_* is the weighted value of the class label of *x_i_* in KNN. For a binary classification between MRSA and MSSA samples, the positive and negative training instances were represented as *n*-dimensional vectors with class labels 1 and 0, respectively. The testing data without class labels are classified into 1 or 0 based on the *k* nearest training samples. In the learning of KNNs classifier, various values of *k* were examined by nested cross validation in order to find the best performance.

**Support Vector Machine (SVM)**

This study was involved in a binary classification between MRSA and MSSA spectra. The positive (MRSA) and negative (MSSA) spectra were labeled with ＋1 and －1, respectively, for the two classes. The training dataset is $X=\left\{x^{t}, c^{t}\right\}$ where *c^t^* = ＋1 if *x^t^* ∈ positive dataset and *c^t^* = －1 if *x^t^* ∈ negative dataset. This work wants to identify *w* and *w*_0_ such that

$$w^{T}x^{t}+w_{0}\geq+1 \text{for} c^{t}=+1 \text{and} w^{T}x^{t}+w_{0}\leq-1 \text{for} c^{t}=-1$$

which can be rewritten as

$c^{t}(w^{T}x^{t}+w_{0})\geq+1$.

This problem could be induced to find out an optimal separating hyperplane that can maximize the margin between two classes [5]. The distance of *x^t^* to the discriminating hyperplane is

$$\frac{|w^{T}x^{t}+w_{0}|}{||w||}$$

and we would like the distance to be higher than a specific value *h*:

$\frac{c^{t}(w^{T}x^{t}+w_{0})}{||w||}\geq h, \forall t and c^{t}\in\{+1,-1\}$.

The support vector machine (SVM) was an advanced algorithm used to identify a hyperplane between two classes with maximum margin based on n-dimensional vector space[5]. With an attempt to maximize *h*, however, an unlimited number of possible values could be elucidated by tuning *w*. Hence, the *h*‖*w*‖ was defined as one and try to minimize ‖*w*‖ by using following solution [6]:

$$\min\frac{1}{2}||w||^{2} subject to c^{t}(w^{T}x^{t}+w_{0})\geq+1, \forall t$$

In this work, SVM could be adopted to determine a hyperplane for discriminating between MRSA and MSSA samples with maximal margin in a vector space containing n dimensions (size of attribute set). The mass-to-charge ratio values of spectra were represented as a numeric vector in an n-dimensional vector space, which are the input values for SVM. A famous SVM public resource, called LIBSVM [7], was downloaded and installed in our computing server for an iterative training of multiple SVMs in accordance with various feature sets. In the machine learning problem, it has been demonstrated that if the best discriminant is nonlinear, instead of enabling a nonlinear modeling, we could map all n-dimensional vectors to new vector space with higher dimension m, where m > n, based on using nonlinear kernel functions. As demonstrated in previous methods [8-11], the radial basis function (RBF) was typically chosen as the specified kernel function on learning of SVM models. The RBF function was given as follows:

$$K(x^{t},x)=\exp\{-\frac{||x^{t}-x||^{2}}{2s^{2}}\}$$

where *x^t^* is the center and *s* is the radius, which should be provided by programmer. When using LIBSVM, cost (*c*) and gamma (*r*) are two supporting parameters used to optimize the radius of kernel function and softness of hyperplane, respectively. To achieve the feasible values of gamma (*r*) and cost (*c*) in model learning, the nested cross validation was adopted in this study.

**Supplementary Figures**
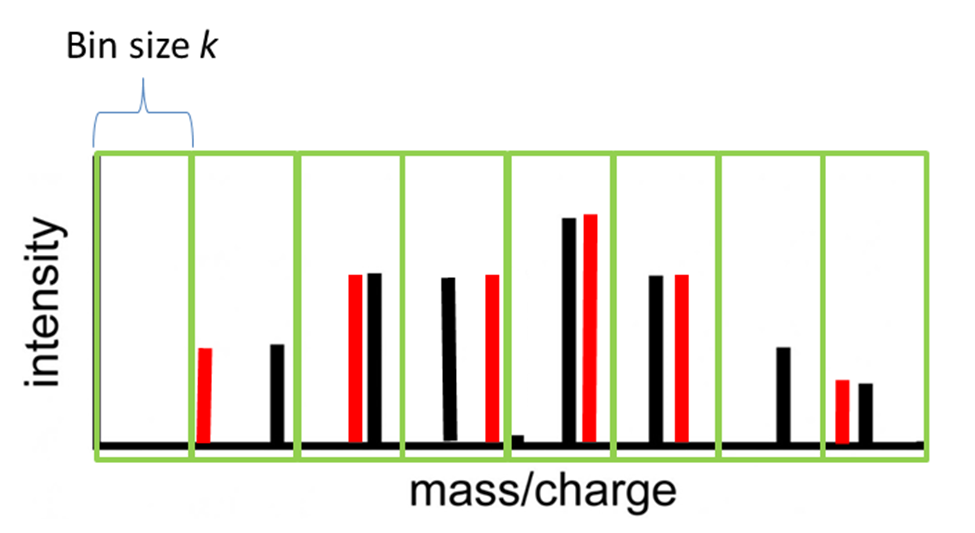


**Figure S1. Schematic diagram of binning method.** Given a specified bin size *k*, ranging from 1 to 15 Da, there are two spectrums marked in different colors (red and black), and the peaks locating within the same bin are considered as the same attribute, e.g. peak 2410~2420 Da (*k* = 10).


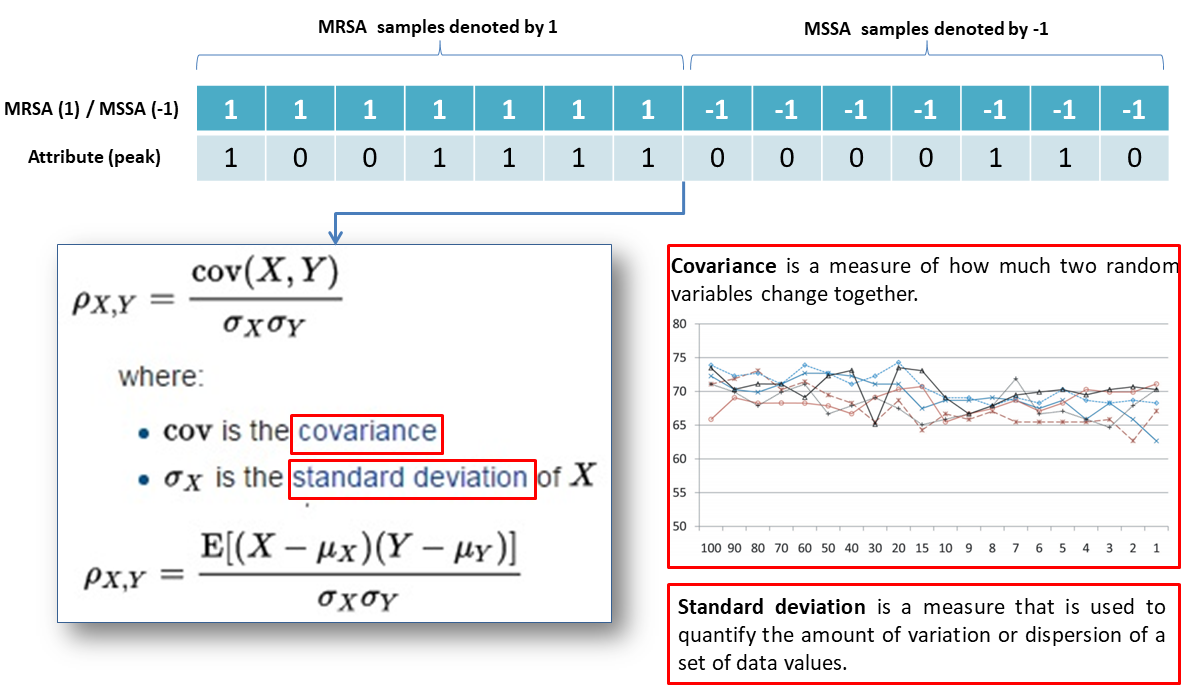


**Figure S2. Schematic diagram for calculating PCC value of each attribute (peak).** Herein, all the positive (MRSA) and negative (MSSA) samples of training dataset are labeled as +1 and -1, respectively. Given an attribute (peak), the samples with/without adequate intensity are labeled as 1/0. After the PCC calculation, an attribute having a higher PCC value indicates that it has a higher correlation to the distribution of positive and negative samples.

**
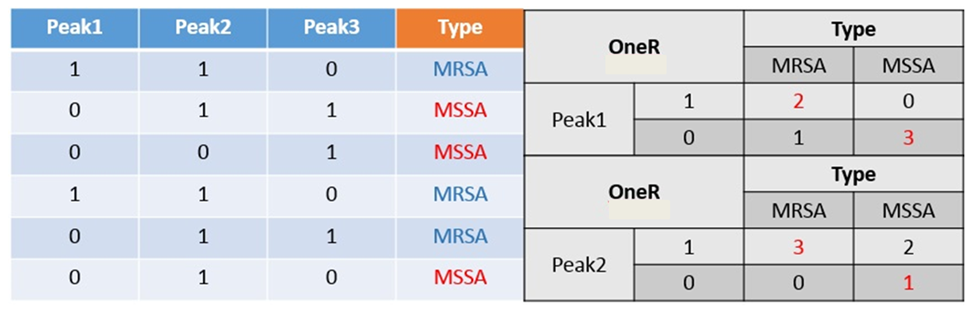
**

**Figure S3. Example of OneR attribute evaluation.** Firstly, a frequency table is constructed for each predictor (single rule) against the target samples. Three independent attributes, peak1, peak2, and peak3, were adopted to generate frequency tables one by one. In this case, each attribute has two kinds of value: 1 (exist) and 0 (not exist). All data will be calculated. The rule for peak1 is defined as: if peak1 exists, the predicted result is completely MRSA; otherwise, the predicted result is MRSA, with one mistake. The error rate for peak1 is 0.167 (one mistake over total six instances). After calculating error rate for each attribute, all of the evaluated attributes were ranked according to their error rate in ascending order. The attribute containing lowest error rate represents a best discriminating power.


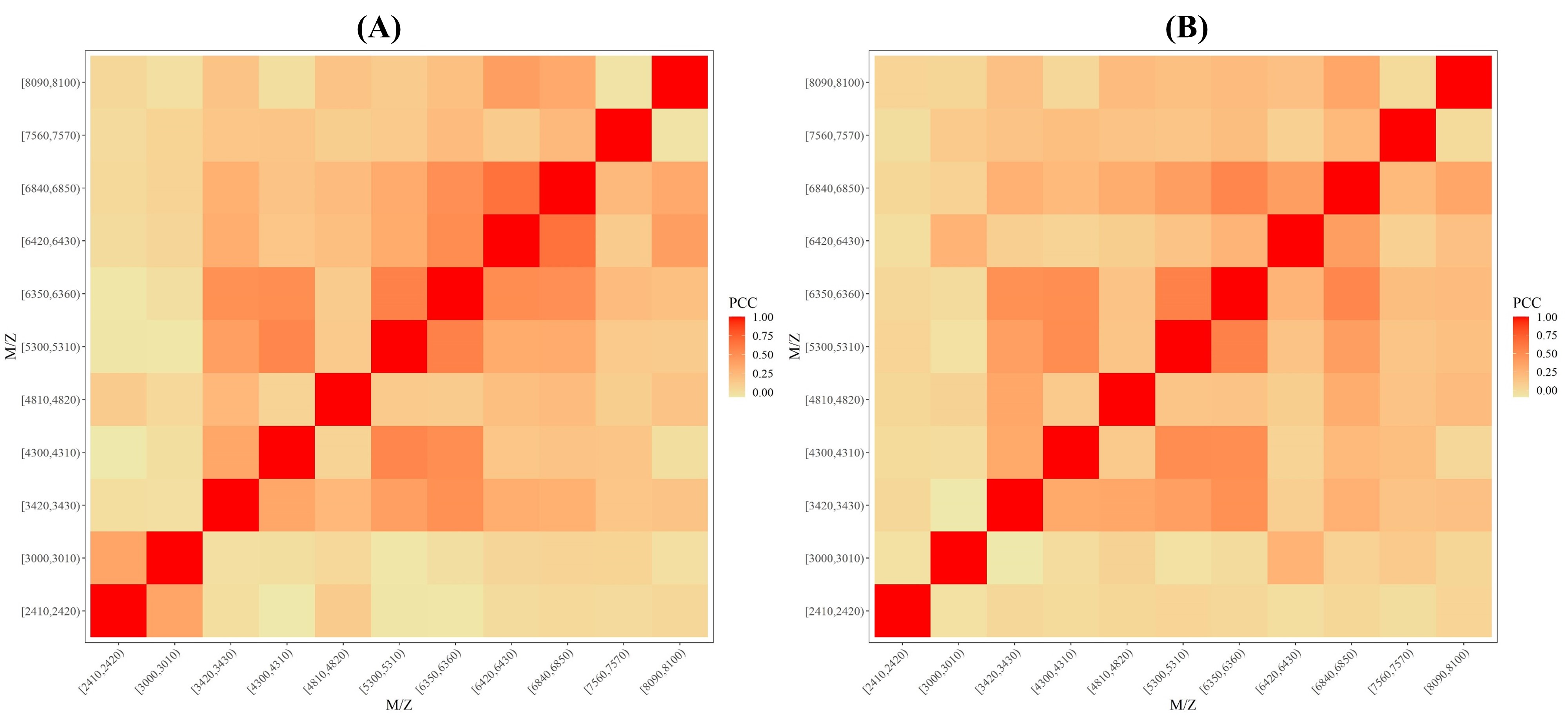


**Figure S4. Correlated peaks investigation for MRSA (A) and MSSA (B) groups, respectively.** The values in the heatmap were the Pearson’s correlation coefficients. These peaks were the informative peaks.

**Supplementary Tables**

**Table S1. The parameters used for nested cross validation for each machine learning methods.**

| **Machine learning method** | **Parameters** | **Meanings*** |
| --- | --- | --- |
| **DT** | minsplit | The minimum number of data that must exist in a node for a split. |
|  | minbucket | The minimum number of observations in any leaf node. |
|  | cp | Any split that does not decrease the overall lack of fit by a factor of cp is not attempted. |
|  | maxdepth | The maximum depth of any node of the final tree, with the root node counted as depth 0. |
| **RF** | num.trees | Number of trees. |
|  | mtry | Number of variables to possibly split at in each node. |
|  | min.node.size | Minimal node size. |
|  | sample.fraction | Fraction of observations to sample. |
| **KNN** | k | Number of neighbors considered. |
|  | kernel | Kernel to use. |
| **SVM** | kernel | Kernel to use. |
|  | gamma | Parameter needed for all kernels except linear. |
|  | cost | The ‘C’-constant of the regularization term in the Lagrange formulation. |

*The meanings of DT, RF, KNN, and SVM quoted from comprehensive R archive network (CRAN) instructions of ‘rpart’, ‘ranger’, ‘kknn’, and ‘e1071’.

**Table S2. Illustration of peak list for bin size 10 Da.** This rank list was generated according to the absolute value of Pearson correlation coefficient.

| **Rank** | **Peak range** | **Pearson correlation coefficient** |
| --- | --- | --- |
| 1 | 2410~2419 | 0.3696 |
| 2 | 2430~2439 | 0.2807 |
| 3 | 6590~6599 | 0.2611 |
| 4 | 5280~5279 | -0.2500 |
| 5 | 2450~2459 | 0.2492 |
| 6 | 6900~6909 | -0.2413 |
| 7 | 6430~6439 | -0.2354 |
| 8 | 6520~6529 | -0.2187 |
| 9 | 2870~2879 | 0.2129 |
| 10 | 3450~3459 | -0.2126 |
| 11 | 10460~10469 | -0.1994 |
| 12 | 5000~5009 | -0.1888 |
| 13 | 6560~6569 | -0.1737 |
| 14 | 2540~2549 | 0.1701 |
| 15 | 2380~2389 | 0.1665 |
| 16 | 6420~6429 | 0.1586 |
| 17 | 5520~5529 | 0.1580 |
| 18 | 11550~11559 | -0.1563 |
| 19 | 7030~7039 | -0.1445 |
| 20 | 2390~2399 | 0.1399 |

**Table S3. Prediction performance of top 15 models.** The rank was according to the AUC obtaing from the 5-fold cross validation. Note that the values are the average and standard deviation of the 5-fold cross validation, respectively. In addition, the parameters of the classifier were adjusted by the nested cross validation firstly, then the nested cross validation was used again for determining the optimal feature sets.

| **Rank** | **Classifier** | **Bin size** | **Order** | **SEN** | **SPE** | **ACC** | **MCC** | **AUC** |
| --- | --- | --- | --- | --- | --- | --- | --- | --- |
| 1 | RF | 7 | COR | 0.8156±0.0073 | 0.8159±0.0075 | 0.8158±0.0074 | 0.6308±0.0148 | 0.8997±0.0046 |
| 2 | RF | 12 | COR | 0.8213±0.0079 | 0.8210±0.0085 | 0.8212±0.0082 | 0.6416±0.0163 | 0.8996±0.0072 |
| 3 | RF | 7 | OneR | 0.8139±0.0097 | 0.8140±0.0102 | 0.8140±0.0100 | 0.6272±0.0199 | 0.8984±0.0039 |
| 4 | RF | 12 | OneR | 0.8179±0.0081 | 0.8178±0.0076 | 0.8179±0.0078 | 0.6351±0.0156 | 0.8977±0.0066 |
| 5 | RF | 15 | COR | 0.8066±0.0072 | 0.8070±0.0071 | 0.8068±0.0072 | 0.6129±0.0143 | 0.8955±0.0056 |
| 6 | RF | 15 | OneR | 0.8150±0.0030 | 0.8146±0.0027 | 0.8149±0.0029 | 0.6290±0.0057 | 0.8952±0.0057 |
| 7 | RF | 14 | OneR | 0.8162±0.0073 | 0.8159±0.0078 | 0.8161±0.0075 | 0.6314±0.0150 | 0.8947±0.0072 |
| 8 | RF | 14 | COR | 0.8139±0.0051 | 0.8134±0.0053 | 0.8137±0.0052 | 0.6266±0.0103 | 0.8944±0.0056 |
| 9 | RF | 8 | COR | 0.8139±0.0036 | 0.8134±0.0035 | 0.8137±0.0036 | 0.6266±0.0071 | 0.8943±0.0038 |
| 10 | RF | 8 | OneR | 0.8122±0.0075 | 0.8121±0.0076 | 0.8122±0.0075 | 0.6237±0.0151 | 0.8932±0.0039 |
| 11 | RF | 6 | COR | 0.8026±0.0125 | 0.8025±0.0119 | 0.8026±0.0122 | 0.6045±0.0244 | 0.8931±0.0033 |
| 12 | RF | 11 | COR | 0.8156±0.0125 | 0.8159±0.0125 | 0.8158±0.0125 | 0.6309±0.0250 | 0.8929±0.0061 |
| 13 | RF | 5 | OneR | 0.8077±0.0045 | 0.8083±0.0039 | 0.8080±0.0042 | 0.6153±0.0084 | 0.8927±0.0088 |
| 14 | RF | 6 | OneR | 0.8049±0.0052 | 0.8045±0.0046 | 0.8047±0.0049 | 0.6087±0.0099 | 0.8923±0.0041 |
| 15 | RF | 11 | OneR | 0.8088±0.0085 | 0.8083±0.0081 | 0.8086±0.0083 | 0.6165±0.0166 | 0.8921±0.0058 |
| 16 | RF | 10 | COR | 0.8100±0.0061 | 0.8102±0.0057 | 0.8101±0.0059 | 0.6195±0.0118 | 0.8921±0.0058 |

Note. RF = Random forest; PCC = Pearson’s correlation coefficient; OneR = One rule attribute evaluation; SEN = Sensitivity; SPE = Specificity; ACC = Accuracy; MCC = Matthew’s correlations coefficient; AUC = Area under the receiver operating characteristic curve.

**Table S4. Prediction performance of considering intensity when bin size is 10 on 5-fold cross validation.**

| **Feature selection** | **Classifier** | **SEN** | **SPE** | **ACC** | **MCC** | **AUC** |
| --- | --- | --- | --- | --- | --- | --- |
| PCC | DT | 0.7036±0.0109 | 0.8255±0.016 | 0.7609±0.0075 | 0.5305±0.0158 | 0.7999±0.0148 |
|  | RF | 0.8043±0.0132 | 0.8038±0.0125 | 0.8041±0.0129 | 0.6075±0.0257 | 0.8853±0.0074 |
|  | KNN | 0.7511±0.0180 | 0.7509±0.0183 | 0.7510±0.0181 | 0.5014±0.0362 | 0.8315±0.0125 |
|  | SVM | 0.7641±0.0101 | 0.7643±0.0100 | 0.7642±0.0100 | 0.5278±0.0201 | 0.8449±0.0197 |
| OneR | DT | 0.7054±0.0564 | 0.7994±0.0567 | 0.7496±0.0084 | 0.5074±0.0132 | 0.7963±0.0201 |
|  | RF | 0.7986±0.0082 | 0.7994±0.0083 | 0.7990±0.0082 | 0.5973±0.0165 | 0.8819±0.0083 |
|  | KNN | 0.7308±0.0195 | 0.7306±0.0187 | 0.7307±0.0191 | 0.4607±0.0382 | 0.8248±0.0193 |
|  | SVM | 0.7494±0.0113 | 0.7490±0.0114 | 0.7492±0.0113 | 0.4978±0.0226 | 0.8306±0.0174 |

Note. PCC = Pearson’s correlation coefficient; OneR = One rule attribute evaluation; DT = decision tree; RF = random forest; KNN = K-nearest neighbors algorithm; SVM = support vector machine; SEN = Sensitivity; SPE = Specificity; ACC = Accuracy; MCC = Matthew’s correlations coefficient; AUC = Area under the receiver operating characteristic curve.

**Table S5. Correlated peaks investigation for peak in [2410,2420) in MRSA and MSSA groups, respectively.** The number (percentage) of spectra which peaks with (without) adequate intensities in both [2410,2420) and other ranges simultaneously. In addition, the correlations were also calculated.

|  | **MRSA** | | | | | | **MSSA** | | | | | |
| --- | --- | --- | --- | --- | --- | --- | --- | --- | --- | --- | --- | --- |
| **Peak range 2** | **With adequate intensities in both ranges** | | | **Without adequate intensities in both ranges** | | **PCC** | **With adequate intensities in both ranges** | | | **Without adequate intensities in both ranges** | | **PCC** |
| [3000,3010) | 469 | (26.53) | 527 | | (29.81) | 0.3638 | 6 | (0.38) | 580 | | (36.94) | -0.0522 |
| [3420,3430) | 421 | (23.81) | 178 | | (10.07) | 0.0066 | 15 | (0.96) | 181 | | (11.53) | 0.0169 |
| [4300,4310) | 437 | (24.72) | 80 | | (4.52) | -0.0660 | 15 | (0.96) | 106 | | (6.75) | 0.0023 |
| [4810,4820) | 473 | (26.75) | 126 | | (7.13) | 0.1187 | 16 | (1.02) | 66 | | (4.20) | 0.0213 |
| [5300,5310) | 414 | (23.42) | 144 | | (8.14) | -0.0488 | 16 | (1.02) | 161 | | (10.25) | 0.0343 |
| [6350,6360) | 418 | (23.64) | 134 | | (7.58) | -0.0497 | 15 | (0.96) | 165 | | (10.51) | 0.0143 |
| [6420,6430) | 459 | (25.96) | 83 | | (4.69) | 0.0170 | 12 | (0.76) | 249 | | (15.86) | -0.0245 |
| [6840,6850) | 467 | (26.41) | 69 | | (3.90) | 0.0302 | 16 | (1.02) | 74 | | (4.71) | 0.0226 |
| [7560,7570) | 406 | (22.96) | 237 | | (13.40) | 0.0236 | 12 | (0.76) | 269 | | (17.13) | -0.0204 |
| [8090,8100) | 401 | (22.68) | 269 | | (15.21) | 0.0390 | 15 | (0.96) | 297 | | (18.92) | 0.0329 |

Note. MRSA = Methicillin-resistant *Staphylococcus aureus*; MSSA = Methicillin-sensitive *Staphylococcus aureus*; PCC = Pearson’s correlation coefficient.

**References**

1. Markey MK, Tourassi GD, Floyd CE, Jr. Decision tree classification of proteins identified by mass spectrometry of blood serum samples from people with and without lung cancer, Proteomics 2003;3:1678-1679.

2. Therneau TM, Atkinson EJ. An introduction to recursive partitioning using the RPART routines. Technical Report 61. URL http://www. mayo. edu/hsr/techrpt/61. pdf, 1997.

3. Liang S-Y, Wu S-W, Hsien Pu T et al. An Adaptive Workflow Coupled with Random Forest Algorithm to Identify Intact N-glycopeptides Detected from Mass Spectrometry. 2014.

4. Liaw A, Wiener M. Classification and regression by randomForest, R news 2002;2:18-22.

5. Vapnik VN. An overview of statistical learning theory, IEEE Trans Neural Netw 1999;10:988-999.

6. Byvatov E, Schneider G. Support vector machine applications in bioinformatics, Appl Bioinformatics 2003;2:67-77.

7. Chang C-C, Lin C-J. Libsvm, ACM Transactions on Intelligent Systems and Technology 2011;2:1-27.

8. Kumari B, Kumar R, Kumar M. PalmPred: an SVM based palmitoylation prediction method using sequence profile information, PLoS One 2014;9:e89246.

9. Lu CT, Chen SA, Bretana NA et al. Carboxylator: incorporating solvent-accessible surface area for identifying protein carboxylation sites, J Comput Aided Mol Des 2011;25:987-995.

10. Chang WC, Lee TY, Shien DM et al. Incorporating support vector machine for identifying protein tyrosine sulfation sites, J Comput Chem 2009;30:2526-2537.

11. Chu C-H, Huang C-M, Liang H-K et al. KinasePhos 2.0: a web server for identifying protein kinase-specific phosphorylation sites based on sequences and coupling patterns, Nucleic Acids Research 2007;35:W588-W594.
